# Broiler physiological response to low phosphorus diets at different stages of production

**DOI:** 10.1016/j.psj.2022.102351

**Published:** 2022-11-19

**Authors:** Adewunmi O. Omotoso, Henry Reyer, Michael Oster, Steffen Maak, Siriluck Ponsuksili, Klaus Wimmers

**Affiliations:** ⁎ Research Institute for Farm Animal Biology (FBN), 18196 Dummerstorf, Germany; † Faculty of Agricultural and Environmental Sciences, University of Rostock, 18059 Rostock, Germany

**Keywords:** broiler chicken, dietary mineral depletion, mineral homeostasis, nutritional conditioning, vitamin D metabolism

## Abstract

Phosphorus (**P**) inclusion in broiler diets needs to meet the physiological demands at a specific developmental stage to ensure the performance, health, and welfare of the birds and minimize nutrient losses. Toward a more efficient utilization of P in broiler husbandry, a timed nutritional conditioning strategy might enhance the endogenous mechanisms of mineral homeostasis and thus reduce dietary P supply of mineral sources. In this study, following a variable P supply in the starter phase, the effects of a dietary P depletion of broiler chickens were investigated at different developmental stages. Physiological adaptation mechanisms were elucidated based on zootechnical performance, endocrine parameters, regulation of intestinal P transport, bone characteristics, and health aspects. The results revealed a marked response to P depletion at the earliest developmental phase, after which indications of effective compensatory mechanism were detectable with advancing ages. Potential mechanisms that enable broilers to maintain mineral homeostasis primarily include endocrine control mediated by calcitriol actions, as well as intestinal P uptake and mineral mobilization from the bone. Conclusively, the precise timing, duration, and extent of a P depletion strategy in the broiler chicken might be considered for optimized nutrient utilization.

## INTRODUCTION

The comprehensive realization of the genetic potential of broiler chickens requires the provision of optimal environmental conditions, which prioritizes a well-founded nutritional regimen specifically with regard to mineral supply, including phosphorus (**P**) and calcium (**Ca**). The inclusion of P as a dietary macromineral in broiler farming is essential to drive various physiological processes. These comprise bone mineralization and integrity, acid-base balance, phospholipid and nucleotide formation, nerve function and cellular energy metabolism (**ATP**), which are critical to the sustenance of growth, productivity and overall welfare of the bird (Proszkowiec-Weglarz and Angel, 2013).

In practice, the amount of the bioavailable P in plant-based diets fed to broilers usually is insufficient, mainly due to the limited or lacking production of endogenous mucosal phosphatases required to facilitate intestinal P release. Therefore, inorganic P sources and exogenous phytases are obligatory to compensate for the low rate of digestible P obtained from cereals and other plant-based feed (Singh, 2008; Dersjant-Li et al., 2015). Nutritional strategies regarding broiler dietary P need to avoid, on the one hand, excessive P supplementation resulting in increased P excretion with the risk of environmental pollution and, on the other hand, avoid undersupply, which might negatively impact development, performance and health (Campbell et al., 2017; Tay-Zar et al., 2019). Hence, P supplementation in broiler diets needs to consider the birds' physiological demands at a specific developmental stage. In fact, adequate P supply in early development is critical for bone mineralization and body growth, prompting the need for high dietary mineral intake in the early stages (Rama Rao et al., 2003; Coto et al., 2008). During the maturation of the bird, the supply of P and administered phytase levels make an important contribution to the maintenance of physiological processes, including muscle differentiation, lipid metabolism and immune functions (Li et al., 2016; Schmeisser et al., 2017; Nari et al., 2020).

Therefore, life-time responses to nutritional conditioning for P efficiency in the broiler and its age-specific demand have shown that broilers fed depleted P at the starter phase manifested adaptive mechanisms of maintaining P homeostasis to support adequate bone mineralization and the modulation of other physiological attributes. The nutritional studies conducted by Yan et al. (2005), in which broilers were subjected to P depletion from d 19 of life, showed efficient P utilization and unchanged weight development in depleted birds compared to controls throughout their life-time, but compensatory actions at the bone level. Furthermore, in an experimental design on broiler chickens with P depletion and P repletion, there was an effect on zootechnical traits during P depletion, which was compensated during P repletion, suggesting a capacity to compensate for an initial depleted mineral status in broiler chickens (Letourneau-Montminy et al., 2008). As for the presumed mechanism, depleted P levels have been reported to stimulate the expression of genes encoding the transcellular P transport in the intestine to drive rapid recovery and equilibrium for the limited mineral supply (Yan et al., 2007; Proszkowiec-Weglarz and Angel, 2013; Lederer, 2014).

Endocrinal regulators such as parathyroid hormone (**PTH**) and calcitriol (1,25(OH)_2_ vitamin D3) act on respective responsive organs, including the intestine, kidney and bone to maintain mineral homeostasis (Bergwitz and Jüppner, 2010; Proszkowiec-Weglarz and Angel, 2013). PTH prevents hypocalcaemia and acts on bone to mobilize Ca and P and reduces high serum P levels by promoting renal P excretion (Blaine et al., 2014). Moreover, PTH acts on calcitriol regulation, which in turn increases mineral absorption in the small intestine and reduces PTH expression in the parathyroid glands (Demay et al., 1992; Brenza et al., 1998). Thus, the interaction of PTH and calcitriol enables the regulation of systemic Ca and P levels via sophisticated feedback loops in the organismal biosystem. Due to variable dietary P supply, the broiler executes compensatory mechanisms by regulating serum calcitriol levels and transcellular P transport in the small intestine via variable abundances of sodium/phosphate co-transporters (Rousseau et al., 2016; Hu et al., 2018). The P retention and body reserves play a critical role in the timing and success of maintaining mineral homeostasis. The broiler's compensatory adaptation to variable P-supply, which incorporates hormonal, transcriptional and bone interactions, must be examined over all developmental stages (starter, grower, and finisher) to achieve economic growth rates with lower P supply in diets.

We hypothesize that the depletion of dietary P supply and its timing contribute to endogenous adaptive responses for P efficiency during the productive life of the broiler and its age-specific requirements. Comprehensive phenotyping which commences immediately post-hatch until market weight with P supply below, equal to or above current recommendations in the starter phase and its subsequent reduction within the groups in grower and finisher phases will identify limits and opportunities for the efficient use of mineral P in broiler chicken farming. The objective of the present study was to evaluate the effects of a variable P supply throughout the entire production phases via measurements for growth performance, endocrine control, transcellular P transport, bone mineralization, and health aspects in an array of tissues such as blood, jejunum, kidney, and bone.

## MATERIALS AND METHODS

### Ethical Statement

The study was approved by the Scientific Committee of the Research Institute of Farm Animal Biology (FBN), and the experimental setup was generally licensed by the ethics committee of the state Mecklenburg-Western Pomerania, Germany (LALLF MV 7221.3-1-051/16).

### Broiler Chickens, Housing, Experimental Diets, and Design

**Figure 1:**
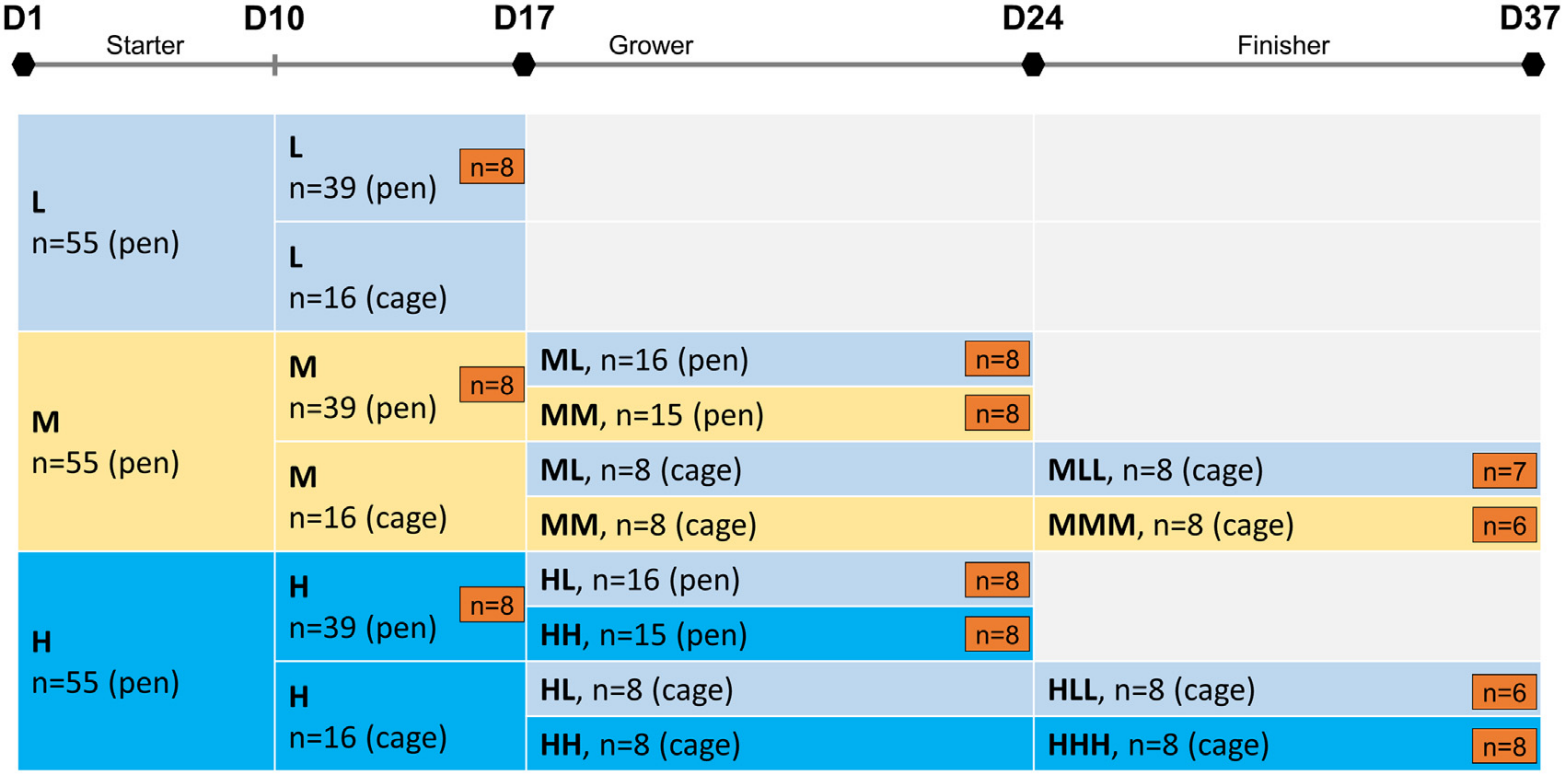
Experimental design. Broiler chickens received 1 of 3 experimental diets containing lower (L), medium (M), or higher (H) levels of dietary P / non-phytate P (nPP) between d 1 and 17. Following an initial dietary assessment during the starter phase, a depletion strategy using a low dietary P level was applied during the grower (ML vs. MM; HL vs. HH) and finisher stages (MLL vs. MMM; HLL vs. HHH). The feeding trial included (i) animals housed in pens until d 24 and (ii) animals housed in individual cages until d 37. A subset of broiler chickens (n = 6–8) were sampled on d 17, 24, and 37 to obtain blood, jejunum, kidney, and bone samples as indicated by orange rectangles.

The study was conducted at a poultry research facility and comprised Ross 308 broiler hatchlings of both sexes (n = 165). Hatchlings with an average body weight of 41.2 g were obtained from WIMEX Agrarprodukte GmbH (Regenstauf, Germany) and were raised on wood shavings as litter material in pens of 3.8 m^2^ per dietary group. At any phase, the animal density in pens was below 25 kg/m^2^, which assured that the current organic standards for broiler space requirements were fulfilled. Each pen was equipped with nipple drinkers and feeders for unrestricted access to water and feed. Lighting and temperature followed recommendations throughout starter (d 1–10; duration: 20 h; intensity: 20 lux; temperature: 30–35°C), grower (d 11–24; duration: 17 h; intensity: 20 lux; temperature: 30–35°C), and finisher phases (d 25–37; duration: 17 h; intensity: 20 lux; temperature: 30–35°C) (Aviagen, 2018). Broiler chickens have been subjected to an oral vaccination against Newcastle Disease at d 9 of life. Broilers were distributed in a completely randomized design where birds received a wheat-corn-soybean meal-based diet without phytase supplementation to minimize exogenous phytase activity. Diets were formulated without the addition of non-starch polysaccharide enzymes. All diets were fed in pelleted form and were formulated according to nutrient recommendations (GfE, 1999) except for P (Tables 1 and 2). This resulted in 3 dietary groups with recommended (M; according to Ross, 2014), lower (L; −50%), or higher (H; +50%) amounts of non-phytate P (**nPP**) fed during the starter developmental stage. The level of soluble phosphorus was quantified in the eluate. Phytase activity was analyzed spectrophotometrically with an LOD of 180 FTU/kg (EN ISO 30024). Birds were randomly assigned to 1 of 3 dietary groups of 55 animals each (Figure 1). For the grower stage at d 11, a subset of sex-balanced broiler chickens were transferred into single cages (45 cm × 45 cm × 45 cm) equipped with nipple drinkers and feeders to record individual feed intake and body weight (Figure 1). Animals in the cages had visual contact with their conspecifics. The assignment to the respective dietary group, that is, M or H, was maintained to ensure adaptation to the new housing environment and grower feed. From d 17, broiler chickens kept in pens as well as those in cages were subjected to a dietary P depletion. Thus, broiler chickens were offered lowered dietary P levels in grower (ML, HL) and finisher phases (MLL, HLL) compared to the starter phase (Figure 1). Accordingly, the experimental design also included non-depleted control groups for the respective stages, that is, MM and HH for grower and MMM and HHH for finisher phases. Animals in pens were kept until d 24, whereas those in individual cages were kept until d 37. Due to high losses among the chickens that received the L diet in the starter phase, the trial was continued only with animals of the M and H groups (details are presented in the results section). Individual body weight of broiler chickens was recorded on d 10, d 17, d 24, and d 37 of life to capture the respective developmental phases.

**Table 1:** Composition of the experimental diets for broiler chickens at starter, grower, and finisher phases.

		Starter (d 1–10)			Grower (d 11–24)			Finisher (d 25–37)		
Ingredient	Unit	−50% nPP	100% nPP	+50% nPP	−50% nPP	100% nPP	+50% nPP	−50% nPP	100% nPP	+50% nPP
Wheat	%	30.0	30.0	30.0	32.0	32.0	32.0	33.5	33.5	33.5
Soybean meal (44% CP)	%	26.0	26.0	26.0	27.0	27.0	27.0	24.0	24.0	24.0
Corn, pre-treated^1^	%	19.4	19.4	19.4	21.0	21.0	21.0	24.0	24.0	24.0
Soybean concentrate (64% CP)	%	11.0	11.0	11.0	6.5	6.5	6.5	5.0	5.0	5.0
Soybean oil	%	5.4	5.4	5.4	6.3	6.3	6.3	6.3	6.3	6.3
Calcium carbonate	%	1.69	1.16	0.63	1.37	0.94	0.48	1.28	0.87	0.40
Cellulose powder	%	1.24	0.60	-	1.10	0.50	-	1.10	0.60	-
Corn starch, pre-gelatinized	%	1.1072	1.1172	1.0472	0.6573	0.7173	0.6273	0.8674	0.8374	0.8874
Brewer's dried yeast	%	1.0	1.0	1.0	1.0	1.0	1.0	1.0	1.0	1.0
Vitamin & trace element premix^2^	%	1.0	1.0	1.0	1.0	1.0	1.0	1.0	1.0	1.0
Monocalcium phosphate, (23% P)	%	0.44	1.60	2.80	0.33	1.30	2.35	0.27	1.21	2.23
Salt, NaCl	%	0.43	0.43	0.43	0.43	0.43	0.43	0.43	0.43	0.43
Choline Cl (50%)	%	0.39	0.39	0.39	0.37	0.37	0.37	0.35	0.35	0.35
DL-Methionine	%	0.35	0.35	0.35	0.34	0.34	0.34	0.29	0.29	0.29
Calcium propionate	%	0.3	0.3	0.3	0.3	0.3	0.3	0.3	0.3	0.3
Lysine HCl	%	0.21	0.21	0.21	0.21	0.21	0.21	0.22	0.22	0.22
Manganese sulphate (33% Mn)	%	0.028	0.028	0.028	0.028	0.028	0.028	0.028	0.028	0.028
Zinc sulphate (36% Zn)	%	0.01	0.01	0.01	0.01	0.01	0.01	0.01	0.01	0.01
Copper sulphate (24% Cu)	%	0.004	0.004	0.004	0.004	0.004	0.004	0.004	0.004	0.004
Vitamin D3 (500.000 IU/g)	%	0.00078	0.00078	0.00078	0.0007	0.0007	0.0007	0.0006	0.0006	0.0006
Threonine	%	-	-	-	0.05	0.05	0.05	0.05	0.05	0.05
ME	kcal/kg	2,988	2,988	2,988	3,059	3,059	3,059	3,107	3,107	3,107
Sum	%	100	100	100	100	100	100	100	100	100
Calcium	%	1.04	1.04	1.04	0.90	0.90	0.90	0.84	0.84	0.84
Phosphorus, total	%	0.51	0.78	1.05	0.47	0.69	0.93	0.44	0.65	0.88
Phosphorus, nPP^3^	%	0.26	0.52	0.78	0.23	0.45	0.68	0.21	0.42	0.64

1 Corn, pre-treated – hydrothermal treatment.

2 Vitamin & trace element premix (SNIFF Spezialdiäten GmbH, Soest, Germany) provided per kg of feed: vitamin A (retinyl acetate), 15.000 IE; vitamin D3 (cholecalciferol), 1.100 IE; vitamin E (all-rac-alpha-tocopheryl acetate), 100 mg; vitamin K3 (menadione), 7 mg; Fe (as FeSO4), 100 mg; Zn (as ZnSO4), 50 mg; Mn (as MnSO4), 30 mg; Cu (as CuSO4), 5 mg; Se (as Na2SeO3), 0.1 mg; I (as Ca(IO3)2), 2.0 mg.

3 nPP, non-phytate phosphorus.

**Table 2:** Wet-chemical analysis of the broiler chicken diets (g/kg as fed basis).

		Starter (d 1–10)			Grower (d 11–24)			Finisher (d 25–37)		
Ingredient	Unit	−50% nPP^1^	100% nPP	+50% nPP	−50% nPP	100% nPP	+50% nPP	−50% nPP	100% nPP	+50% nPP
Dry matter	g/kg	908	907	909	903	904	904	905	903	902
Crude protein	g/kg	243	248	243	225	217	221	201	203	198
Crude ash	g/kg	59	63	65	55	59	62	50	53	57
Crude fat	g/kg	55	63	61	48	48	58	68	68	68
Sucrose	g/kg	50.7	51.5	52.2	53.8	52.5	52.2	53	52.4	53.3
Total starch	g/kg	347	347	354	365	363	362	397	393	399
Calcium	g/kg	10	9.8	9.9	8.7	9.1	9.2	7.9	8.1	8.4
Phosphorus, total	g/kg	4.7	7.2	9.5	4.3	6.4	8.6	4	6.1	8.1
Phosphorus, soluble	g/kg	2.5	3.7	4.9	2.5	3.5	4.6	2.2	3.4	4.4
Magnesium	g/kg	2.1	2	2	1.8	1.9	1.9	1.8	1.7	1.7
Potassium	g/kg	9.7	9.8	9.9	9.0	9.2	9.3	8.5	8.5	8.5
Sodium	g/kg	1.8	1.7	1.8	1.8	2.1	2	1.7	1.8	1.8
ME	kcal/kg	2,892	2,988	2,988	2,844	2,820	2,892	3,059	3,035	3,035
Phytase activity^2^	FTU/kg	211	<180	314	267	218	<180	267	194	199

1 nPP, non-phytate phosphorus.

2 Phytase activity was analyzed spectrophotometrically with an LOD of 180 FTU/kg (EN ISO 30024).

### Serum and Tissue Sample Collection

A total of 83 broiler chickens were sampled at the 3 sampling stages, that is, d 17 (n = 24 from pens), d 24 (n = 32 from pens), d 37 (n = 27 from cages) as outlined in Figure 1. Sampling comprised 4 birds per sex per dietary group at the grower sampling stages (d 17, d 24) and at least 3 birds per sex per dietary group at the finisher sampling stage (d 37). At the respective growth stages, birds were randomly selected, anaesthetized by electrical stunning, and slaughtered between 09h00 and 12h00. Trunk blood was collected in anticoagulant-free tubes and allowed to clot for 20 min. Serum was prepared by centrifugation at 3500 × *g* for 15 min. Serum samples were stored at −80°C until further analysis. Furthermore, sections of jejunal tissue (∼3 cm in length) were collected proximal to the Meckel's Diverticulum. Jejunal samples were rinsed with a 0.9% NaCl solution, snap-frozen in liquid nitrogen and stored at −80°C until RNA extraction. Moreover, the right kidneys were sampled, snap-frozen in liquid nitrogen and stored at −80°C until RNA extraction. Finally, the right femurs of the birds were collected and stored at −0°C until further analysis.

### Serum Minerals and Hormones Measurement

Serum samples were analyzed to determine calcium, inorganic P, and albumin levels using commercial assays for the Fuji DriChem 4000i device according to the manufacturer's instructions (FujiFilm, Minato, Japan). In addition, hormone measurements were prepared in duplicate and measured using commercially available enzyme-linked immunosorbent assay (**ELISA**) kits. Corresponding kits for ELISA were processed according to the manufacturer's instructions for PTH (CSB-E11880Ch, CUSABIO, Houston, TX), triiodothyronine (EIA-4569, DRG, Marburg, Germany), calcidiol (EIA-5396, DRG), and calcitriol (IDS-AC-62F1, Immunodiagnostic Systems, Frankfurt am Main, Germany). The raw data was processed according to 4-parameter logistic curve analysis.

### Bone Breaking Strength and Ash

Femur samples were thawed overnight at room temperature. Individual bones were weighed, and the length, width, and maximum diameter (epiphysis) of the femur samples were measured to ascertain linear bone growth. The bone-breaking strength (force) was estimated at the calculated midpoint (50% of length) using a 3-point bending/flexural test device (WINOPAL Forschungsbedarf, Elze, Germany). The proximal and distal epiphyses of individual femur was placed horizontally on the 2 supporting anvils of the bending test device. Relative to the length of the femurs an adjusted diaphyseal free span (fulcrum point) ranging between 2.0 and 3.0 cm was set. The vertical loading anvil with a capacity of 50 kg was then applied to the mid-diaphysis of each bone at a speed of 2 mm/s until the bone failed. A computerized monitor recorded the load-displacement curve illustrating the estimated fracture load. Moreover, the ash percentage of femora was analyzed. Separated diaphyseal regions of the femur from the prior bending test were homogenized with a high-speed grinder. Approximately 1.5 g of homogenized samples were transferred in triplicates to pre-heated and weighed porcelain crucibles for incineration at 600°C for 7 h in the muffle furnace. Afterwards, samples were left overnight at 105°C in the drying cabinet. Samples were charred on the Bunsen burner and incinerated for 7 h in the muffle furnace at 600°C. The resultant material was cooled at room temperature. After adding a few drops of 30% hydrogen peroxide (H_2_O_2_), samples were transferred to the drying cabinet (105°C for 1 h). Samples were returned to the muffle furnace for incineration at 600°C for 10 min. Afterward, bone ash was allowed to cool in the desiccator for approximately 30 min followed by final weight measurement on a precision scale. The bone ash percentage was calculated (ash% = final weight / initial weight × 100).

### RNA Isolation, Purification, and cDNA Synthesis

Prior to RNA extraction, snap-frozen jejunal, and renal samples were transferred into a sterile ceramic mortal placed in a liquid nitrogen bath and pulverized with a pestle to homogenize. Subsequently, total RNA was isolated from the pulverized samples using TRI Reagent with adherence to the manufacturer's guidelines (Sigma-Aldrich, Taufkirchen, Germany). DNase I was used for DNA digestion followed by purification with column-based NucleoSpin RNA II-Kit (Macherey-Nagel, Düren, Germany). RNA concentration was determined using the NanoDrop ND-2000 spectrophotometer (Thermo Fisher Scientific, Dreieich, Germany). Further integrity test was done by visualization via agarose gel electrophoresis. The presence of genomic DNA contamination was checked by polymerase chain reaction (**PCR**) amplification of the chicken *GAPDH* gene using an intron-spanning primer set (forward primer: 5^'^- AGTCGGAGTCAACGGATTTG -3′; reverse primer: 5′ -CTGCCCATTTGATGTTGCTG- 3′). Subsequently, cDNA was synthesized using 1,500 ng of RNA, together with random primers (Promega, Mannheim, Germany), oligo d(T) nucleotides, and RNAsin plus (Promega), in the presence of SuperScript III Reverse Transcriptase (Invitrogen, Karlsruhe, Germany) according to the manufacturer's instructions. The cDNA samples were diluted with *Aqua dest*. to a final volume of 200 µL and stored at −20°C. Further verification for the absence of genomic DNA in the synthesized cDNA was conducted with another PCR for chicken *GAPDH* employing SupraTherm Taq DNA polymerase (GeneCraft, Münster, Germany).

### Quantitative Real-Time PCR

To assess the contribution of transcellular P transporters in the kidney and intestine, gene expression of solute carrier family 20 and solute carrier family 34 members were analyzed. Presently, there are 4 known candidate genes encoding chicken transcellular P transporters (Table 3), namely; solute carrier family 20 member 1 (***SLC20A1***)*, s*olute carrier family 20 member 2 (***SLC20A2***)*, s*olute carrier family 34 member 1 (***SLC34A1***), and solute carrier family 34 member 2 (***SLC34A2***). Gene expression analysis was conducted using these 4 genes alongside β-actin (***ACTB***) as a housekeeping gene, to quantify the transcript abundance via real-time PCR assay. The gene-specific primers, corresponding annealing temperatures, and resulting fragment lengths are stated in Table 4. Individual cDNA samples were analyzed in duplicate on the LightCycler 480 System (Roche, Mannheim, Germany). Reactions were performed in a final volume of 12 µL comprising Light Cycler 480 SYBR Green I Master mix (Roche) and gene-specific primers. The temperature profiles included an initial denaturation step at 95°C for 5 min, followed by 45 cycles comprising denaturation at 95°C for 10 s, annealing at the specific temperature for 15 s, and extension/fluorescence acquisition at 72°C for 25 s. The quality and specificity of amplified products were assessed by the melting curve analysis. For all assays, threshold cycles were converted to copy numbers of respective transcripts using standard curves generated by amplifying serial dilutions of the corresponding PCR standard (10^7^ to 10^1^ copies). Transcript copy numbers were factorial normalized based on *ACTB* expression values and log2 transformed prior to further analysis.

**Table 3:** List of genes encoding annotated sodium/phosphate co-transporters in broiler chickens.

Gene symbol	Aliases	Ensembl ID	Gene name
*SLC20A1*	*GLVR1, PiT-1*	ENSGALG00000013740	Solute carrier family 20 member 1
*SLC20A2*	*GLVR-2, Ram-1, PiT-2*	ENSGALG00000038336	Solute carrier family 20 member 2
*SLC34A1*	*NaPi-2a, NPT2a, NaPi-IIa*	ENSGALG00000003075	Solute carrier family 34 member 1
*SLC34A2*	*NaPi-2b, NPT2b, NAPi-IIb*	ENSGALG00000014372	Solute carrier family 34 member 2

**Table 4:** Gene-specific primers used for mRNA expression analysis via RT-qPCR.^1^

Gene symbol	Primer sequence (5’-3′)	Melting temperature (°C)	Amplicon length (bp)
*SLC20A1*	FOR: CTCTCGTCGTCTGGTTCTTTG	60	95
	REV: CTTCTCCATCAGCGGACTTTC	60	
*SLC20A2*	FOR: TGCTGCTACCATTGCTATTAACG	60	161
	REV: TTCTCTTCATCCAGGGGCATAC	60	
*SLC34A1*	FOR: CTTTTGCTGGTGCTACAGTGC	61	167
	REV: CGTGATGATTTTCAGCAGGTC	61	
*SLC34A2*	FOR: CTGATCTTGCCATCGGTCTC	60	170
	REV: TCCAGCCAGCCAAGTAAAAG	60	
ACTB*	FOR: CCTCTTCCAGCCATCTTTCTT	60	254
	REV: TAGAGCCTCCAATCCAGACA	60	

Abbreviations: *ACTB*, beta-actin; FOR, Forward; REV, Reverse; *SLC20A1*, solute carrier family 20 member 1; *SLC20A2*, solute carrier family 20 member 2; *SLC34A1*, solute carrier family 34 member 1; *SLC34A2*, solute carrier family 34 member 2.

1 Primers used for the expression analysis were designed using the Primer-BLAST software on the NCBI platform (https://www.ncbi.nlm.nih.gov/tools/primer-blast/).

⁎ Housekeeping gene.

### Statistical Data Analysis

The experimental design comprised 3 dietary P groups (L, M and H), with a subsequent dietary P depletion (ML, HL, MLL, HLL) and their controls (MM, HH, MMM, HHH) across the 3 distinct developmental stages (starter, grower, finisher) between 2 sexes of Ross 308 broilers raised under 2 housing conditions (individual cages, pens). The body weight and feed intake for the broiler chickens at the respective growth stages were measured and used to determine the body weight gain and feed conversion ratio (**FCR**). Traits were analyzed within each phase using the linear model: γ_ij_ = μ + m_i_ + n_j_ + β_W_ + ε_ij_, where γ_ij_ are the measurements of the response variable (i.e., zootechnical traits, bone traits, serum traits and gene expression), μ represents the overall mean, m_i_ represents effect of the dietary P group, n_j_ represents sex effect, β_W_ is the linear effect of the covariates individual body weight or femur weights as stated in the result section and ε_ij_ represents the residual error. Mortality rates per diet group per phase were analyzed for association via Fisher's Exact Test. Analyses were performed using the R package stats and lmerTest (R core team, 2019; package; Kuznetsova et al., 2017). The pairwise comparison of means between dietary groups was achieved with the embedded Tukey post-hoc test. Differences were considered as statistically significant at *P* ≤ 0.05.

## RESULTS

In this study, the effects of dietary P depletion were investigated at 3 developmental stages throughout the productive life span, starting with a variable P supply immediately after hatching. The feeding regimen included P supply below, at, or above current recommendations early in life and subsequent P reduction within these groups. Responses of broilers were ascertained via performance, serum metabolites, bone parameters, and mRNA expression of transcellular sodium/phosphate co-transporters.

### Zootechnical Parameters

Based on an average body weight of 41.2 g at d 1, the dietary treatments within the starter phase resulted in a body weight of 204 ± 3 g (L; n = 62; mean ± SE), 311 ± 4 g (M; n = 60), and 312 ± 4 g (H; n = 59) at d 10, which is statistically significant in the comparison between L and both M and H groups. Data for chickens kept in individual cages from day 10 onwards is presented in Table 5. At the early grower phase (d 10–17), L fed broiler chickens kept in cages showed significantly reduced body weight, feed intake, weight gain, and FCR compared to other dietary groups (Table 5). For body weights at d 17, similar results were obtained from birds kept in pens regarding group L (n = 29; 354 ± 14 g), M (n = 35; 748 ± 11 g), and H (n = 35; 769 ± 13 g). The L group showed a significantly increased mortality in the phase from d 1 to 17 (31%) compared to M (5%) and H groups (7%). With advancing development, that is, the grower phase (d 17–24), feed intake, and mortality revealed no significant differences between the dietary groups for individual cages (Table 5) and pens for groups ML (n = 13; 1,308 ± 21 g), HL (n = 13; 1,333 ± 41 g), MM (n = 15; 1,266 ± 30 g), and HH (n = 14; 1,352 ± 46 g). Body weight and body weight gain significantly differed between the depleted P diet and control groups (Table 5). In addition, chickens of the ML group revealed significantly higher FCR than the other groups. Finally, at the finisher phase (d 24–37), zootechnical data such as body weight, feed intake, FCR, and mortality were unaltered between dietary groups (Table 5).

**Table 5:** Body weight, feed intake, body weight gain and feed conversion ratio (FCR) of broiler chickens housed in individual cages and fed divergent amounts of dietary P throughout experimental phases. All values are displayed as mean ± SE.

Phase	Diet (n)	Body weight (g)	Feed intake (g)	Body weight gain (g)	FCR (g/g)
d 10	L (n = 16)	226 ± 4^b^			
	M (n = 16)	335 ± 6^a^			
	H (n = 16)	332 ± 4^a^			
d 10–17	L (n = 10)	377 ± 15^b^	229 ± 14^b^	147 ± 17^b^	1.73 ± 0.19^a^
	M (n = 16)	613 ± 14^a^	406 ± 18^a^	278 ± 13^a^	1.49 ± 0.08^ab^
	H (n = 16)	629 ± 18^a^	396 ± 16^a^	298 ± 17^a^	1.35 ± 0.03^b^
d 17–24	ML (n = 8)	1093 ± 41^b^	646 ± 39	480 ± 23^c^	1.35 ± 0.07^a^
	HL (n = 8)	1136 ± 52^ab^	627 ± 27	528 ± 25^bc^	1.19 ± 0.02^b^
	MM (n = 7)	1201 ± 52^ab^	670 ± 30	586 ± 33^ab^	1.15 ± 0.02^b^
	HH (n = 8)	1252 ± 55^a^	687 ± 36	601 ± 38^a^	1.15 ± 0.02^b^
d 24–37	MLL (n = 7)	2171 ± 145	1858 ± 158	1086 ± 71^b^	1.80 ± 0.12
	HLL (n = 6)	2159 ± 199	1852 ± 271	1177 ± 115^ab^	1.94 ± 0.13
	MMM (n = 6)	2450 ± 188	2021 ± 210	1254 ± 68^ab^	1.69 ± 0.08
	HHH (n = 8)	2632 ± 94	2244 ± 79	1436 ± 39^a^	1.63 ± 0.03

Abbreviations: H, high P diet; HH, high-high P diet; HL, high-low P diet; HLL, high-low-low P diet; HHH, high-high-high P diet; L, low P diet; M, medium P diet; ML, medium-low P diet; MM, medium-medium P diet; MLL, medium-low-low P diet; MMM, medium-medium-medium P diet.

a-c Column-wise disparity of superscripts indicates statistical significance (*P* < 0.05) between dietary P groups within phase.

### Serum Mineral and Hormone Measurements

**Figure 2:**
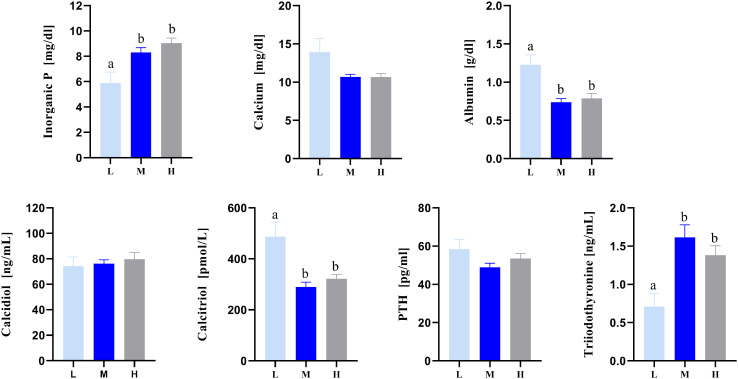
Serum parameters of broiler chickens fed divergent amounts of dietary P until 17 d of life. Values are displayed as means ± SE. Superscripts indicate statistical significance (*P* <  0.05) between the dietary groups. PTH, parathyroid hormone; L, low P diet (n = 8); M - medium P diet (n = 8); H, high P diet (n = 8).

**Figure 3:**
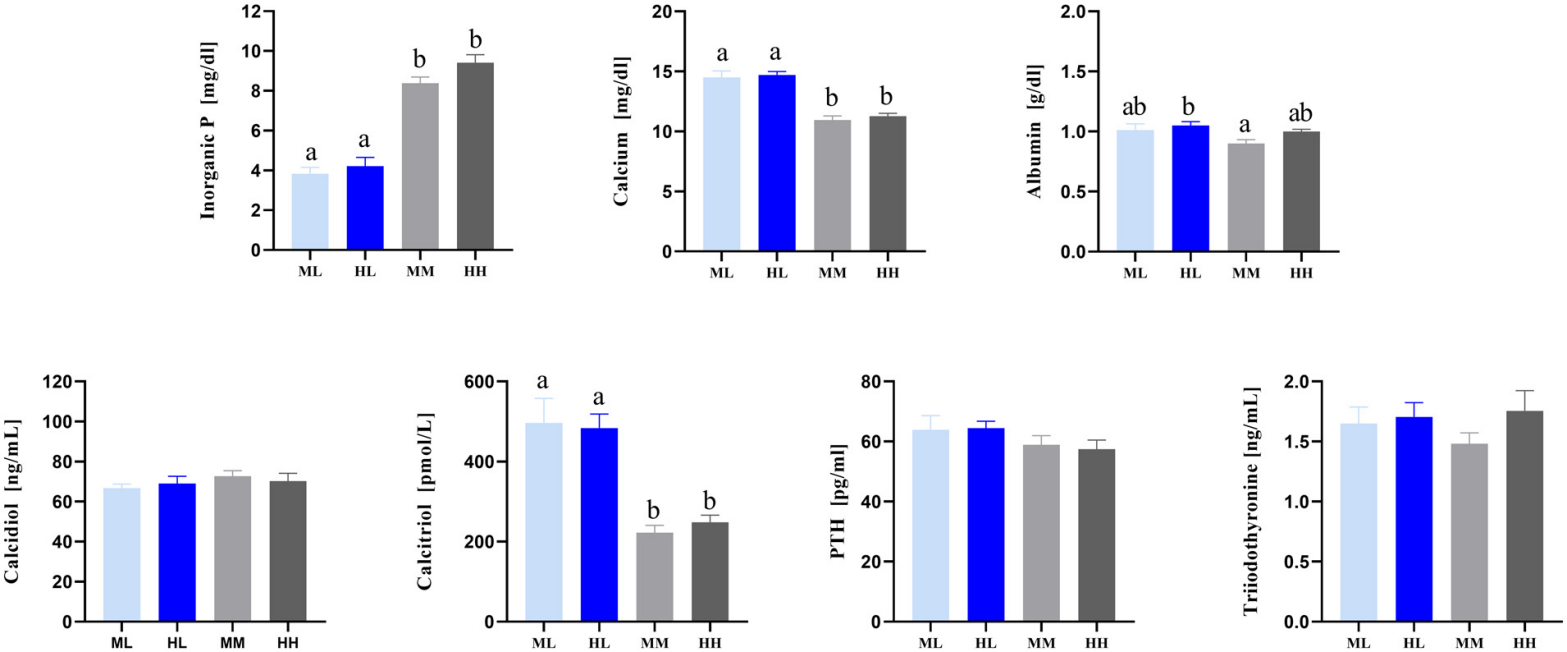
Serum parameters of broiler chickens fed divergent amounts of dietary P until 24 d of life. Values are displayed as means ± SE. Superscripts indicate statistical significance (*P* <  0.05) between the dietary groups. PTH, parathyroid hormone; ML, medium-low P diet (n = 8); MM, medium-medium P diet (n = 8); HL, high-low P diet (n = 8); HH, high-high P diet (n = 8).

**Figure 4:**
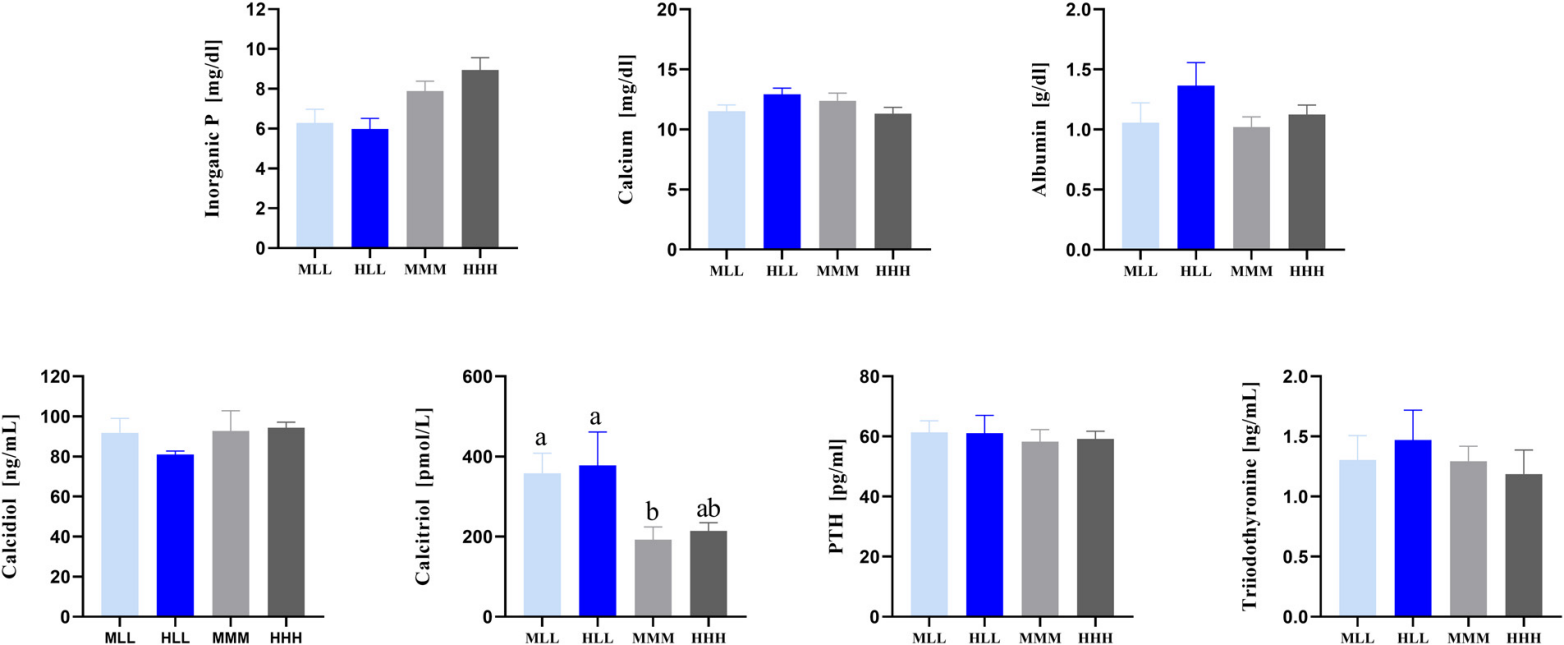
Serum parameters of broiler chickens fed divergent amounts of dietary P until 37 d of life. Values are displayed as means ± SE. Superscripts indicate statistical significance (*P* <  0.05) between the dietary groups. PTH, parathyroid hormone; MLL - medium-low-low P diet (n = 7); MMM, medium-medium-medium P diet (n = 6); HLL, high-low-low P diet (n = 6); HHH, high-high-high P diet (n = 8).

On d 17, L fed broiler chickens showed significantly reduced serum levels of P and triiodothyronine (T3) and significantly increased levels of albumin and calcitriol compared to those fed M and H diets (Figure 2). On d 24, significant differences were observed in serum levels of P, calcium, albumin, and calcitriol (Figure 3). The P depleted groups (ML, HL) showed reduced serum P levels but increased levels of calcium, albumin, and calcitriol compared with the control groups (MM, HH). On d 37, serum calcitriol levels differed significantly between P depleted groups (MLL, HLL) and animals fed MMM, but not compared to broilers fed HHH (Figure 4). Concentrations of calcidiol and PTH were unaffected by diet at all experimental stages.

### Bone Trait Measurement

On d 17, L fed broiler chicken significantly differed for bone traits (force, weight, diameter, and ash) compared to those fed M and H diets (Table 6). On d 24, significant differences were observed in the bone fracture load of broilers fed the P depleted groups (ML, HL) and those fed MM. Analyses of other bone traits such as bone weight, length, diameter, and ash analyses showed no diet-dependent differences. On d 37, broiler chickens fed depleted P (MLL, HLL) significantly differed from those fed the MMM and HHH diet for breaking force. Femora bone diameter differed significantly between broilers fed the depleted P (MLL), and broilers fed the MMM and HHH. Values for bone weight, length, and ash remained unaffected.

**Table 6:** Femoral bone traits of broiler chickens fed divergent amounts of dietary P throughout experimental phases. All values are displayed as mean ± SE.

Day of sampling	Diet (n)	†Fracture load (N)	Weight (g)	‡Length (cm)	‡Diameter (cm)	‡Ash (%)
d 17	L (n = 8)	30.9 ± 4.23^a^	1.9 ± 0.09^a^	4.0 ± 0.08^a^	0.5 ± 0.02^a^	7.1 ± 0.24^a^
	M (n = 6)	231.1 ± 13.19^b^	3.9 ± 0.27^b^	5.2 ± 0.11^b^	0.6 ± 0.02^b^	15.2 ± 0.60^b^
	H (n = 7)	211.3 ± 9.91^b^	3.8 ± 0.21^b^	5.2 ± 0.07^b^	0.7 ± 0.02^b^	16.1 ± 0.64^b^
d 24	ML (n=8)	199.7 ± 7.73^ac^	6.8 ± 0.32	6.4 ± 0.05	0.8 ± 0.01	18.4 ± 2.05
	HL (n = 8)	214.7 ± 13.17^c^	6.8 ± 0.32	6.3 ± 0.08	0.8 ± 0.03	18.0 ± 1.64
	MM (n = 6)	293.4 ± 42.60^b^	6.7 ± 0.45	6.3 ± 0.08	0.8 ± 0.03	17.2 ± 1.38
	HH (n = 7)	253.3 ± 22.34abc	6.5 ± 0.51	6.2 ± 0.14	0.8 ± 0.04	20.8 ± 1.09
d 37	MLL (n = 7)	192.8 ± 16.79^ab^	10.5 ± 0.98^ab^	7.5 ± 0.20	0.9 ± 0.04^a^	16.1 ± 0.79
	HLL (n = 6)	176.7 ± 15.28^b^	9.8 ± 0.60^a^	7.3 ± 0.16	0.9 ± 0.02^ab^	16.1 ± 0.85
	MMM (n = 5)	259.2 ± 43.06^c^	11.3 ± 1.46^ab^	7.3 ± 0.19	1.1 ± 0.09^b^	18.5 ± 1.59
	HHH (n = 7)	261.3 ± 11.39^c^	11.8 ± 0.74^b^	7.6 ± 0.13	1.1 ± 0.04^b^	17.6 ± 0.81

Abbreviations: H, high P diet; HH, high-high P diet; HL, high-low P diet; HLL, high-low-low P diet; HHH, high-high-high P diet; L, low P diet; M, medium P diet; ML, medium-low P diet; MM, medium-medium P diet; MLL, medium-low-low P diet; MMM, medium-medium-medium P diet.

† Body weight factored as covariate.

‡ Femoral weight factored as covariate.

a-c Column-wise disparity of superscripts indicates statistical significance (p<0.05) between dietary P groups within phase.

### Temporal Gene Expression of Jejunal and Renal Transcellular Phosphorus Transporters

On d 17, divergent P diets elicited significant differences in jejunal and renal mRNA expressions of sodium/phosphate co-transporters in the birds (Table 7). Specifically, increased jejunal expression of *SLC34A2* (L>H; FC= 2.27) was observed in L fed broilers compared to those that received an H diet. In addition, renal mRNA expression of *SLC20A1* significantly increased (M<H; FC = 3.61) in broilers fed the H diet compared to the M diet. Likewise, renal *SLC20A2* transcripts differed between broilers fed the H diet compared to those fed the L diet (L<H; FC = 1.96) and those fed M diets (M<H; FC = 1.99). Renal *SLC34A1* mRNA abundances differed significantly between broilers fed the L and M diet (L>M; FC = 2.04). Furthermore, renal *SLC34A2* mRNA abundance increased (L<H; FC = 1.96) in the animals fed H compared to those fed the L diet. A tissue-specific expression was observed for both sodium/phosphate co-transporter II genes, with *SLC34A2* predominantly expressed in the jejunum and *SLC34A1* in the kidney. On d 24 and 37, jejunal and renal sodium/phosphate co-transporters expression were unaffected between the dietary groups.

**Table 7:** Gene expression levels of sodium/phosphate co-transporters expressed in jejunum and kidney of broiler chickens fed divergent amounts of dietary P throughout experimental phases. Transcript copy numbers were presented as log2 values (mean ± SE).

Day of sampling	Tissue	Diet (n)	SLC20A1	SLC20A2	SLC34A1	SLC34A2
d 17	Jejunum	L (n = 8)	13.8 ± 0.44	14.4 ± 0.56	8.4 ± 0.91	17.7 ± 0.30^a^
		M (n = 8)	14.2 ± 0.45	13.9 ± 0.61	7.0 ± 0.81	17.0 ± 0.36^ab^
		H (n = 8)	14.0 ± 0.39	13.8 ± 0.59	5.8 ± 0.56	16.5 ± 0.25^b^
	Kidney	L (n = 8)	13.4 ± 0.48^ab^	14.4 ± 0.23^a^	21.2 ± 0.26^a^	8.2 ± 0.20^a^
		M (n = 8)	12.0 ± 0.41^a^	14.4 ± 0.25^a^	20.2 ± 0.29^b^	8.5 ± 0.29^ab^
		H (n = 8)	13.9 ± 0.21^b^	15.4 ± 0.20^b^	20.4 ± 0.19^ab^	9.1 ± 0.23^b^
d 24	Jejunum	ML (n = 8)	12.2 ± 0.97	12.4 ± 0.22	5.8 ± 0.59	16.6 ± 0.37
		HL (n = 8)	9.9 ± 1.93	11.8 ± 0.40	7.3 ± 0.28	15.9 ± 0.49
		MM (n = 8)	10.7 ± 1.45	11.8 ± 0.20	5.6 ± 0.62	16.0 ± 0.31
		HH (n = 8)	11.3 ± 1.02	12.1 ± 0.22	3.9 ± 1.25	16.1 ± 0.24
	Kidney	ML (n = 8)	13.5 ± 0.43	13.4 ± 0.45	20.1 ± 0.69	7.6 ± 0.65
		HL (n = 8)	15.3 ± 1.34	15.9 ± 1.46	23.9 ± 1.31	10.0 ± 1.46
		MM (n = 8)	13.5 ± 0.90	13.7 ± 1.16	19.3 ± 2.33	9.3 ± 1.21
		HH (n = 8)	12.6 ± 0.94	13.7 ± 0.17	20.5 ± 0.44	7.5 ± 0.28
d 37	Jejunum	MLL (n = 7)	13.3 ± 1.09	13.1 ± 0.54	8.5 ± 0.77	16.1 ± 0.82
		HLL (n = 6)	13.0 ± 0.74	11.7 ± 0.99	11.2 ± 0.84	15.5 ± 0.41
		MMM (n = 6)	13.4 ± 0.91	12.2 ± 0.98	9.8 ± 0.78	14.3 ± 0.92
		HHH (n = 8)	13.2 ± 0.65	12.6 ± 0.47	8.3 ± 0.87	16.0 ± 0.82
	Kidney	MLL (n = 7)	14.0 ± 0.58	14.7 ± 0.90	22.0 ± 1.04	9.8 ± 1.30
		HLL (n = 6)	14.5 ± 1.38	14.9 ± 1.18	21.5 ± 1.05	8.9 ± 1.07
		MMM (n = 6)	14.8 ± 0.26	14.3 ± 0.27	19.4 ± 1.05	8.2 ± 0.46
		HHH (n = 8)	15.8 ± 0.86	15.0 ± 0.95	20.8 ± 0.32	9.0 ± 0.96

Abbreviations: H, high P diet; HH, high-high P diet; HL, high-low P diet; HLL, high-low-low P diet; HHH, high-high-high P diet; L, low P diet; M, medium P diet; ML, medium-low P diet; MM, medium-medium P diet; MLL, medium-low-low P diet; MMM, medium-medium-medium P diet.

a-b Column-wise disparity of superscripts indicates statistical significance (*P* < 0.05) between dietary P groups within tissue within phase.

## DISCUSSION

Efficient nutrient utilization in broiler chickens needs to account for endogenous responses to variable P supply to benefit from enhanced P absorption in the intestine, P retention in the bone, and P reabsorption by the kidney. The age-appropriate provision of dietary P is particularly relevant in the early growth phase, when a sufficient amount of dietary P is necessary to meet physiological needs for health and tissue integrity (Baradaran et al., 2021). At the same time, excess dietary P must be prevented due to environmental burden, which has led to the development of feeding strategies tailored to meet age-specific requirements (Abbasi et al., 2019). In the current study, broilers fed the L diet during the early growth phase (d 1–17) had lower body weight and feed intake, while weight gain and FCR increased, accompanied by an increased mortality compared to broilers fed the M or H diets. This suggests that it is only possible to a limited extent to condition a thrifty phenotype with high P efficiency in later life by reducing P supply in early life. This observation contrasts with previous studies that reported broilers raised on dietary nPP levels as low as 0.25% and 1.0% calcium on d 1 to 21 could thrive without microbial phytase supplementation, although weight reductions of up to 15% were observed (Waldroup et al., 2000). It was also reported that dietary P depletion at early and late developmental stages can be applied to reduce P excretion, whereby dietary Ca intake has been shown to be a significant influencing factor (Rousseau et al., 2016). However, the dietary mineral composition applied to birds of the L group in our study (0.26% nPP, 1.04% Ca; Table 1) proved to be insufficient in the early growth phase. The inconsistent observations regarding the potential for P reduction between the studies might be due to the differences in dietary formulations and the interplay of feed components, including varying Ca:P ratios and the level and degradation of plant-based phytase (Sommerfeld et al., 2018). In addition, strain-specific metabolic requirements are also conceivable. In fact, a genetic contribution to P utilization has been shown in chickens and other species, including pigs and quails (Reyer et al., 2019; Vollmar et al., 2020). In addition, the decreased feed intake in broiler chickens raised on the L starter diet may indicate a humoral control of feeding behaviour, although in vertebrates the mechanisms for sensing P by integrating signals from different tissues are still largely unclear (Michigami et al., 2018).

A low P diet impacted bone traits of broilers chickens during each phase, including bone fracture load, weight, length, diameter, and ash, compared to the chickens in the M and H diet groups. The lowered bone mineralization indicates a reduced P availability to drive ossification (Taylor et al., 2013; Shao et al., 2019) or an increased mobilization of bone retained minerals to meet growth and other physiological processes (Li et al., 2020). Although the bone parameters obtained are in a comparable range to recent reports (Süzer et al., 2019; Eusemann et al., 2022), no conclusive statement on optimal mineralization in broilers can be concluded from these values. In the current study, broilers showed femoral sensitivity to P depletion, as P serves as a significant component of the bone due to its deposition in the complex form of hydroxyapatite (Ca_5_(PO_4_)_3_(OH) and is essential for the musculoskeletal development. Reduced feed intake of the L diets compared to M and H diets at d 17 certainly limited the available P pool within the birds resulting in age-inappropriate skeletal development. However, a previous study reported that broiler chickens fed depleted P diets at the grower phase showed similar consequences for bone traits, but also exhibited an increased propensity for bone mineralization processes when mineral deficiencies were replenished later in life (Valable et al., 2018).

Serum P concentrations of broilers fed the L diets until d 17 were lowest compared to broilers that received the M and H diets. This was further triggered due to the reduction in the broilers' feed intake. Contrastingly, serum calcitriol (active vitamin D) levels were highest in broilers fed the L diet compared to broilers fed the M and H diet. Calcitriol plays a pivotal role in the broiler's homeostatic regulation of P by mediating increased intestinal absorption, usually under the conditions of nutritional mineral deficit to attain equilibrium (Berndt and Kumar, 2009). Hence, vitamin D metabolism is essential for adaptation throughout the feeding phases studied to ensure P homeostasis. However, the physiological effect of the endogenous responses to the L diet in the early phase is clearly limited, as broiler chickens showed higher serum albumin and lower serum triiodothyronine (T_3_) concentrations compared to broilers fed the M and H diets. Albumin plays a physiological role in maintaining colloidal osmotic pressure and acts as a marker for renal integrity. Serum levels indicate a severe malnourishment of broilers fed the L diet, suggesting dehydration as well as catabolic metabolism due to the reduction in feed intake (Wadden et al., 1990). In fact, P deprivation has been reported to induce hypothyroidism, and therefore, systemic growth reduction, especially at the early stages of development in the broiler chicken (Parmer et al., 1987; Jianhua et al., 2000). The skeleton represents a target tissue for T_3_, which contributes to the regulation of bone turnover (Williams, 2013). Decreased T_3_ levels are associated with fracture risk and may be related to impaired bone resorption and formation via reduced osteoblast differentiation and function (Vestergaard et al., 2005; Tuchendler and Bolanowski, 2014). Studies in murine bone cells revealed that a higher T_3_ concentration increased bone resorption and made the osteoblasts more sensitive to the actions of PTH (Schmid et al., 1986). Hence, higher serum calcium levels coupled with high levels of T_3_ are probably due to the interactive effects of T_3_ on bone resorption and recruitment of osteoblasts in response to PTH.

Significant differences were observed in serum concentrations of P and Ca between the depleted P groups (ML, HL) and controls (MM, HH) at d 24, indicative of the birds' attempt to achieve mineral homeostasis for both micronutrients with advancing age. Broadly, broilers from the grower phase onwards (d 18–37) exhibited the capacity to tolerate the effects of dietary P for zootechnical properties (Waldroup et al., 2000; Bar et al., 2003). In this context, endocrine control mechanisms represent the adaptive response to address reductions of P supply. Notably, the effect of the serum calcitriol remained elevated in L diet broilers throughout the entire developmental phase, indicating subtle effects such as the continued endocrine mediation of intestinal absorption and renal reabsorption of P (Li et al., 2021).

Dietary effects on other serum metabolites (P, Ca, albumin, calcidiol, PTH, and T_3_) were not present in the depleted P groups and controls at d 37. However, broilers fed L diets at the grower and finisher phases showed effects on bone traits compared to the broilers fed the control and H diets. Although bone fracture load was reduced at d 24, that is after only 1 week on an L diet, the results indicate intact tissue development and absence of pathophysiologic abnormalities. The broiler chickens fed high P diets across developmental phases showed no merits for zootechnical, endocrine, or bone traits compared with birds fed recommended and depleted P levels. It must be noted that numerical differences in the respective traits exist between the experimental groups, which require the validation of the results with a larger sample size. In view of the current results, providing safety margins for dietary P supply do not reveal beneficial outcomes for the bird, but rather exacerbates environmental impacts further associated with the scarcity of P resources (Campbell et al., 2017). This observation is in line with previous findings focusing on high P bioavailability (Gautier et al., 2018).

The jejunum has been reported as the primary site of intestinal P absorption in the broiler (Hurwitz and Bar, 1970). Notably, intestinal P absorption in the broiler occurs via paracellular (passive) and transcellular (active) transport mechanisms. Whereas the former involves the selective molecule diffusion or inhibition through alternative sealing or pore-forming characteristics via the tight-junction protein permeability gradients, for example, claudins (Marks, 2019). The latter entails the recruitment of sodium/phosphate dependent co-transporters located at the brush border membrane (Eto et al., 2006). The transcellular pathway is thought to be the preferential intestinal P absorption route under dietary P restrictions (Marks, 2019). The expression of jejunal *SLC34A2* in response to the diet in the current study suggests that intestinal P availability in the diets affected the abundance of sodium/phosphate co-transporters in jejunal cells to maintain homeostasis and adaptation at the earlier growth phase (Hu et al., 2018). In fact, several studies highlighted an increased expression of genes encoding transcellular P transport in the small intestine of both broiler chickens and laying hens on exposure to deficient P diets, implicating a cellular response to luminal P concentrations via endocrinal factors, for example, calcitriol (Rousseau et al., 2016; Hu et al., 2018; Knöpfel et al., 2019; Proszkowiec-Weglarz et al., 2019; Sommerfeld et al., 2020). In addition, the expression of the *SLC34A1* gene in the kidney of L fed broilers indicated its contributory regulatory role, including reabsorption of P at the proximal tubule to achieve P homeostasis. The renal type III Na^+^-P co-transporters were more abundant in broilers of the H group compared to L and M (SLC20A2) and M group (SLC20A1), respectively. Previous studies on murine models identified responses of the type III Na^+^-P co-transporters to changes in dietary P contents (Candeal et al., 2017; Marks, 2019). Albeit the type III Na^+^-P co-transporters were expressed in response to P levels within the organism's biosystem, suggesting possible transmembrane transport and intracellular utilization, their precise functional potential for P regulation remains unclear (Marks, 2019).

Genes encoding sodium/phosphate co-transporters in the jejunum and kidney remained unaffected throughout the grower and finisher phase (d 18–37), in contrast to observable responses at the endocrine and bone level. This observation suggests that broilers use other compensatory mechanisms to achieve P efficiency in addition to subtle changes in active transcellular P transport in the gut and kidney. In other studies, a low-P diet has not consistently been shown to increase mRNA abundance of transcellular P transporters (Just et al., 2018; Reyer et al., 2021). This could indicate complementary paracellular P transport, post-transcriptional modifications, or miRNA-mediated regulation mechanisms. However, it also broadly informs the broilers' capacity to cope with the P nutrient challenge, for example, depletion, through synergistic interactions of endocrinal and genetic factors towards zootechnical performance and bone traits. Adequate P supply in the early growth phase is thus the basis for long-term physiological P efficiency in the finisher phase (Valable et al., 2020; Baradaran et al., 2021).

In summary, broiler chickens showed physiological responses to different dietary P levels at different developmental stages, as shown by the interplay between serum P and Ca levels, endocrine responses in terms of calcidiol, calcitriol, PTH, and triiodothyronine levels, bone strength and mineralization, and jejunal and renal P transporter gene expressions to maintain P homeostasis for production and welfare. Based on these results, the threshold for P deprivation for environmental concerns should be set no earlier than the late start/early growth phase, as physiological adaptation mechanisms to P deficiency seem more effective than in the early growth phase. In this study, a one-third reduction in P intake in the early growth phase up to d 17 resulted in severe developmental abnormalities that could not be tolerated and compensated. Consequently, nutritional strategies such as the efficient application of phytases and targeted P reduction in the late rearing or early finishing phase are conceivable. The feeding management might be in accordance to the MLL group, as broilers at this age show higher tolerance and faster compensation capacity without compromising production as well as health traits by the adaptation mechanisms investigated in this study.
